# Multi-species sequence comparison reveals conservation of ghrelin gene-derived splice variants encoding a truncated ghrelin peptide

**DOI:** 10.1007/s12020-015-0848-7

**Published:** 2016-01-20

**Authors:** Inge Seim, Penny L. Jeffery, Patrick B. Thomas, Carina M. Walpole, Michelle Maugham, Jenny N. T. Fung, Pei-Yi Yap, Angela J. O’Keeffe, John Lai, Eliza J. Whiteside, Adrian C. Herington, Lisa K. Chopin

**Affiliations:** Comparative and Endocrine Biology Laboratory, Translational Research Institute-Institute of Health and Biomedical Innovation (TRI-IHBI), Queensland University of Technology, Woolloongabba, QLD 4102 Australia; Ghrelin Research Group, Translational Research Institute-Institute of Health and Biomedical Innovation (TRI-IHBI), Queensland University of Technology, Woolloongabba, QLD 4102 Australia; Australian Prostate Cancer Research Centre, Queensland, Princess Alexandra Hospital, Queensland University of Technology, Woolloongabba, QLD 4102 Australia; Molecular Epidemiology Laboratory, QIMR Berghofer Medical Research Institute, Herston, QLD 4006 Australia; Signal Transduction Laboratory, QIMR Berghofer Medical Research Institute, Herston, QLD 4006 Australia

**Keywords:** Ghrelin, Peptide hormone, Evolution, Comparative endocrinology, Alternative splicing

## Abstract

The peptide hormone ghrelin is a potent orexigen produced predominantly in the stomach. It has a number of other biological actions, including roles in appetite stimulation, energy balance, the stimulation of growth hormone release and the regulation of cell proliferation. Recently, several ghrelin gene splice variants have been described. Here, we attempted to identify conserved alternative splicing of the ghrelin gene by cross-species sequence comparisons. We identified a novel human exon 2-deleted variant and provide preliminary evidence that this splice variant and in1-ghrelin encode a C-terminally truncated form of the ghrelin peptide, termed minighrelin. These variants are expressed in humans and mice, demonstrating conservation of alternative splicing spanning 90 million years. Minighrelin appears to have similar actions to full-length ghrelin, as treatment with exogenous minighrelin peptide stimulates appetite and feeding in mice. Forced expression of the exon 2-deleted preproghrelin variant mirrors the effect of the canonical preproghrelin, stimulating cell proliferation and migration in the PC3 prostate cancer cell line. This is the first study to characterise an exon 2-deleted preproghrelin variant and to demonstrate sequence conservation of ghrelin gene-derived splice variants that encode a truncated ghrelin peptide. This adds further impetus for studies into the alternative splicing of the ghrelin gene and the function of novel ghrelin peptides in vertebrates.

**Electronic supplementary material:**

The online version of this article (doi:10.1007/s12020-015-0848-7) contains supplementary material, which is available to authorized users.

## Introduction

Ghrelin is a 28-amino acid peptide hormone produced in the stomach and has potent appetite-stimulating effects [1, 2]. Ghrelin was initially described as a growth hormone-releasing peptide and is now recognised to have a diverse range of functions in a number of cell types and systems, including roles in energy balance, appetite regulation and food-seeking behaviour, insulin regulation and cell proliferation [1–4]. The ghrelin gene (*GHRL*) and the coding exons of the derived preprohormone, preproghrelin, are conserved in a wide range of species including fish [5], turtles [6], birds [7] and mammals [8]. In humans, exons 1 to 4 encode the 117-amino acid preproghrelin, with exons 1 and 2 coding for the 28-amino acid peptide hormone ghrelin [1]. After cleavage of a 23-amino acid signal peptide, proghrelin is processed to form ghrelin, and the third ghrelin residue (serine) is post-translationally octanoylated (acylated) by the enzyme ghrelin *O*-acyl transferase (GOAT, encoded by *MBOAT4*) [9, 10]. The C-terminus of proghrelin (encoded by exons 3 and 4) is further processed to give rise to the 23-amino acid peptide hormone obestatin [11], which has independent actions from ghrelin [12].

We and others have previously demonstrated that the ghrelin gene locus is complex and gives rise to numerous transcripts that could encode a wide range of peptides [13, 14]. A number of ghrelin variants have been characterised in humans [13] and transcripts containing intronic sequence, in1-ghrelin and in2c-ghrelin, have recently been described [15, 16]. Intron 2 cryptic (in2c) ghrelin is an insulin-regulated transcript that contains intron 2-derived exons and the coding region for ghrelin, but lacks the obestatin sequence [16]. The in1-ghrelin transcript contains exon 1, intron 1 and exon 2, and the inclusion of intron 1 leads to a truncation of the ghrelin peptide sequence [15]. We recently reported an exon 2-deleted splice variant in mouse and sheep [17]. In this study, we sought to determine whether any ghrelin gene-derived splice variants are conserved across vertebrates and present preliminary findings on the function of splice variants encoding derived short, C-terminally truncated ghrelin peptides (here termed minighrelin).

## Materials and methods

### Bioinformatics

Ghrelin gene (*GHRL*) sequences (from genomes and transcriptomes) were derived from the UCSC multiway tool, NCBI GenBank [18], and from the NCBI Short Read Archive (SRA) [19]. Putative *GHRL* sequences were interrogated using BLAST [20] in a local instance of the Ruby-based SequenceServer (http://www.sequenceserver.com), gmap v2013-06-27 (a genomic mapping and alignment program for mRNA and EST sequences) with the parameters --cross-species --align --direction=sense_force -Y [21], and custom Perl scripts with BioPerl modules [22]. MUSCLE [23] was used for protein sequence alignments of ghrelin gene orthologs, using the human sequence as the reference.

### Cell culture

Cell lines were originally obtained from the American Type Culture Collection (ATCC, Rockville, MD, USA). The PC3 (ATCC CRL-1435), DU145 (ATCC HTB-81), LNCaP (ATCC CRL-1740) and 22Rv1 (ATCC CRL-2505) prostate cancer cell lines were maintained in Roswell Park Memorial Institute (RPMI) 1640 medium (Invitrogen, Mulgrave, VIC, Australia) with 10 % New Zealand Cosmic Calf Serum (FCS, Thermo Fisher Scientific Australia, Scoresby, VIC, Australia) supplemented with 100 U/mL penicillin G and 100 µg/mL streptomycin (Invitrogen). The non-tumourigenic RWPE-1 (ATCC CRL-11609) and the transformed, tumourigenic RWPE-2 (ATCC CRL-11610) prostate epithelium-derived cell lines were cultured in keratinocyte serum-free medium (KSFM) (Invitrogen) supplemented with 50 µg/mL bovine pituitary extract and 5 ng/mL epidermal growth factor (Invitrogen). All cell lines were passaged at 2- to 3-day intervals at 70 % confluency using TrypLE Select (Invitrogen). Cell morphology and viability were monitored by microscopic observation and regular PCR testing was performed (Universal Mycoplasma Detection Kit, ATCC) to ensure that cells were not contaminated with *Mycoplasma*.

### Identification of human exon 2-deleted preproghrelin

Human exon-2 deleted preproghrelin was cloned as follows: total RNA was harvested from cultured cells using an RNeasy Plus Mini Kit (QIAGEN, Germantown, MD, USA) according to the manufacturer’s instructions. Next, 2 μg total RNA was subjected to DNase I digestion (amplification grade, Invitrogen), immediately followed by cDNA synthesis with SuperScript III using oligo(dT)_18_ primers (Invitrogen) according to the manufacturer’s instructions. RT-PCR primers spanning exon 1 and 3 of preproghrelin (5′-CATGCTCTGGCTGGACTTGG-3′ and 5′-GACAGCTTGATTCCAACATCAAAGG-3′) were designed using PerlPrimer [24]. RT-PCR using tissue (normal human tissue cDNA panel HMRT102 purchased from OriGene, Rockville, MD, USA) and cultured cells was performed in a total reaction volume of 30 μL containing 1× PCR buffer, 0.2 mM deoxynucleotide triphosphates, 1.5 mM MgCl_2_, 0.2 μM primers, 1 μL cDNA and 1 unit of Platinum Taq DNA Polymerase (Invitrogen) on a PTC-200 thermal cycler (MJ Research, Watertown, MA, USA) according to the manufacturer’s instructions, with an annealing temperature of 61 °C. Negative (no-template) controls were performed. RT-PCR products were purified using a MinElute (QIAGEN) PCR Purification Kit, cloned into *pCR4*-*TOPO* (Invitrogen), transformed into One Shot MAX Efficiency DH5α-T1R chemically competent cells (Invitrogen) and sequenced at the Australian Genome Research Facility (AGRF, Brisbane, Australia) using BigDye III (Applied Biosystems, Foster City, CA, USA).

### Food intake as a measure of in vivo function of ghrelin peptides

Acylated (octanoylated) and desacyl 28-AA ghrelin peptides (H-GSSFLSPEHQRVQQRKESKKPPAKLQPR-OH) and 13-AA minighrelin peptides (H-GSSFLSPEHQRVQ-OH) were commercially synthesised (Mimotopes, Melbourne, VIC, Australia). Male 16-week-old C57BL/6J mice, purchased from the Animal Resources Centre (Perth, Western Australia), were housed separately and handled daily for 1 week with unrestricted access to standard chow and drinking water to acclimatise them to experimental conditions. Mice were then injected intraperitoneally with 2 nmol/mouse acyl ghrelin, desacyl ghrelin, acyl minighrelin, desacyl minighrelin, or saline (vehicle) (*n* = 6 per group except for the minighrelin treatment, where *n* = 5). This dose of acyl ghrelin (2 nmol/mouse) has previously been demonstrated to stimulate appetite in mice [25, 26]. Mice were injected at 09:00 (light phase) and given pre-weighed chow pellets. At 4 h post injection, the remaining chow pellets were weighed and the cumulative food intake per mouse was determined to the nearest 0.1 g (pre-injection pellet weight minus post-injection pellet weight). Experiments were carried out with approval of the Animal Ethics Committee, University of Queensland.

### Forced overexpression of human exon 2-deleted preproghrelin

Coding regions of human exon 2-deleted preproghrelin and canonical preproghrelin were commercially synthesised and cloned into OriGene *pCMV6*-*AC* plasmid vectors (Blue Heron Biotechnology, Bothell, WA, USA). Constructs, and empty vector controls, were transformed into *E. coli* DH5α cells (Invitrogen) and purified using a QIAGEN plasmid purification kit, according to the manufacturer’s instructions. To produce a control cell line expressing the vector only, the PC3 prostate cancer cell line was transfected with *pCMV6*-*AC* plasmid DNA using Lipofectamine 2000 reagent (Invitrogen), according to the manufacturer’s instructions. Stably overexpressing PC3 prostate cancer cells were selected with 600 µg/mL G418 antibiotic (Invitrogen). Overexpression of ghrelin variants was confirmed by semi-quantitative RT-PCR (as described above).

### Cell proliferation assays

Cell proliferation assays were performed using the xCELLigence Real-Time Cell Analyzer (RTCA) system (ACEA Biosciences, San Diego, CA, USA), using E plates, according to the manufacturer’s instructions. Briefly, cell lines overexpressing exon 2-deleted preproghrelin, canonical preproghrelin, or empty vector were cultured until they were 70 % confluent, detached from the cell culture flask using trypsin/EDTA (Invitrogen) and collected by centrifugation. Cells were then added to E plates at a density of 5000 cells/well in growth medium with 10 % New Zealand Cosmic Calf serum (FCS) (Thermo Fisher Scientific). Proliferation was measured for up to 72 h and compared to the rate of proliferation of cells expressing an empty vector. Each treatment (exon 2-deleted preproghrelin, canonical preproghrelin and empty vector overexpressing cells) was performed with three replicates, and the experiment was performed independently three times.

### Cell migration assay

Cell migration assays were performed using the xCELLigence RTCA system and cell/invasion migration (CIM) plates with 8 µm pores (ACEA Biosciences). The lower well contained media with 10 % foetal calf serum as a chemoattractant, and the cells were added to the upper well at a density of 50,000 cells/insert in serum-free media. Migration was observed in real time for up to 48 h. Each ‘treatment’ (exon 2-deleted preproghrelin, canonical preproghrelin and empty vector overexpressing cells) was performed with three replicates, and the experiment was performed independently three times.

### Statistical analyses

Statistical analyses were undertaken using GraphPad Prism v5.04 (La Jolla, CA, USA) and R statistical software v3.0.2 (http://www.r-project.org). If a data set did not differ significantly from a normal distribution (*P* ≤ 0.05, Shapiro–Wilk test), the data were considered to have a ‘normal’ distribution and a parametric one-way ANOVA with Tukey’s post hoc test was used to compare groups. If a data set differed significantly from a normal distribution (*P* ≥ 0.05, Shapiro–Wilk test), the Mann–Whitney *U* test (a general non-parametric test for two groups) was utilised to test for a difference between a treatment group and the control.

## Results and discussion

### Evidence of conserved exon skipping of the ghrelin gene

**Fig. 1:**
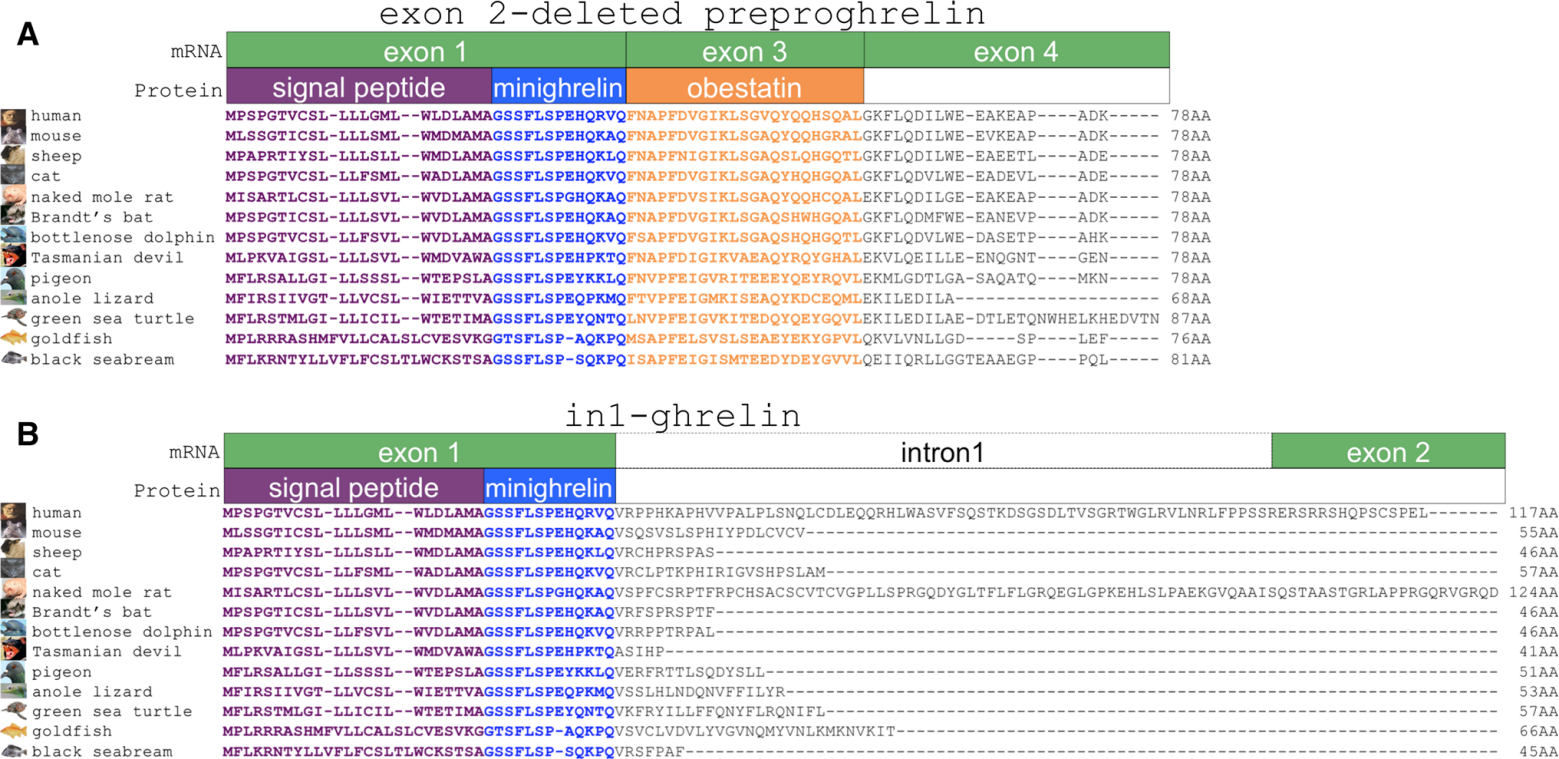
Overview of exon 2-deleted preproghrelin and in1-ghrelin open reading frames in vertebrates. Bioinformatic prediction of **a** exon 2-deleted preproghrelin and **b** in1-ghrelin peptide sequences. Nucleotide databases of diverse species were interrogated for the preproghrelin gene (*GHRL*) sequences. Canonical coding sequences (mRNA) are shown as *green boxes*, while the corresponding regions of the signal peptide (*purple*), minighrelin (*blue*) and obestatin (*orange*) are represented as *boxes* below

We interrogated the ghrelin gene (*GHRL*) in 77 species, representing all major vertebrate groups sequenced to date. This analysis revealed that loss of exon 2 could be tolerated in all taxa. Exon 2 ranges in size from 78 bp in the black seabream teleost fish (*Acanthopagrus schlegeli*) [27] to 117 bp in humans (Online Resource 1). As the length of exon 2 is divisible by three in all species, it is a symmetrical exon. Frequently, skipping of symmetrical exons does not alter the reading frame of the encoded protein [28]. Indeed, the removal of exon 2 from putative preproghrelin mRNAs of all species examined allows the retention of the cognate preproghrelin reading frame. We recently described an exon 2-deleted ghrelin splice variant in mice and sheep [17]. The canonical human preproghrelin coding sequence consists of 117 amino acids (AAs). The 28-AA ghrelin peptide is encoded by exons 1 and 2, while exons 3 and 4 encode the 66-amino acid C-terminal peptide, C-ghrelin, which contains the 23-amino acid peptide obestatin encoded entirely by exon 3 [1, 11]. In contrast, skipping of exon 2 would result in the translation of a 78-AA preprohormone, exon 2-deleted preproghrelin, and would consist of a 13-AA C-terminally truncated ghrelin peptide (hereafter termed minighrelin) that is directly followed by the coding sequence of obestatin (Fig. 1a; Online Resource 2).

In addition to evidence of conserved exon 2 ‘deletion’ (exon skipping), we noted that in1-ghrelin, a splice variant previously cloned from the mouse [29], human and baboon [15], also harbours a minighrelin peptide sequence. In1-ghrelin contains exon 1, the short intron 1 and exon 2 [15]. In contrast to all other described variants (e.g. canonical preproghrelin, in2c-ghrelin, exon 2-deleted preproghrelin and exon 3-deleted preproghrelin), in1-ghrelin lacks exons 3 and 4. In accordance with genome-wide transcriptome analyses [30], we speculate that inclusion of intronic (intron 1 in its entirety) sequence promotes an alternative polyadenylation site within exon 2 or the large intron 2 (~3 kb in human). Due to divergence in the C-terminal region, in1-ghrelin differs greatly in size between species, ranging from 45 AAs in black seabream, 46 AAs in sheep, 55 AAs in mouse, 117 AAs in human, to 124 AAs in the naked mole rat (Fig. 1b). The common feature of in1-ghrelin in all vertebrate species examined is the presence of C-terminally truncated ghrelin sequence, minighrelin.

**Fig. 2:**
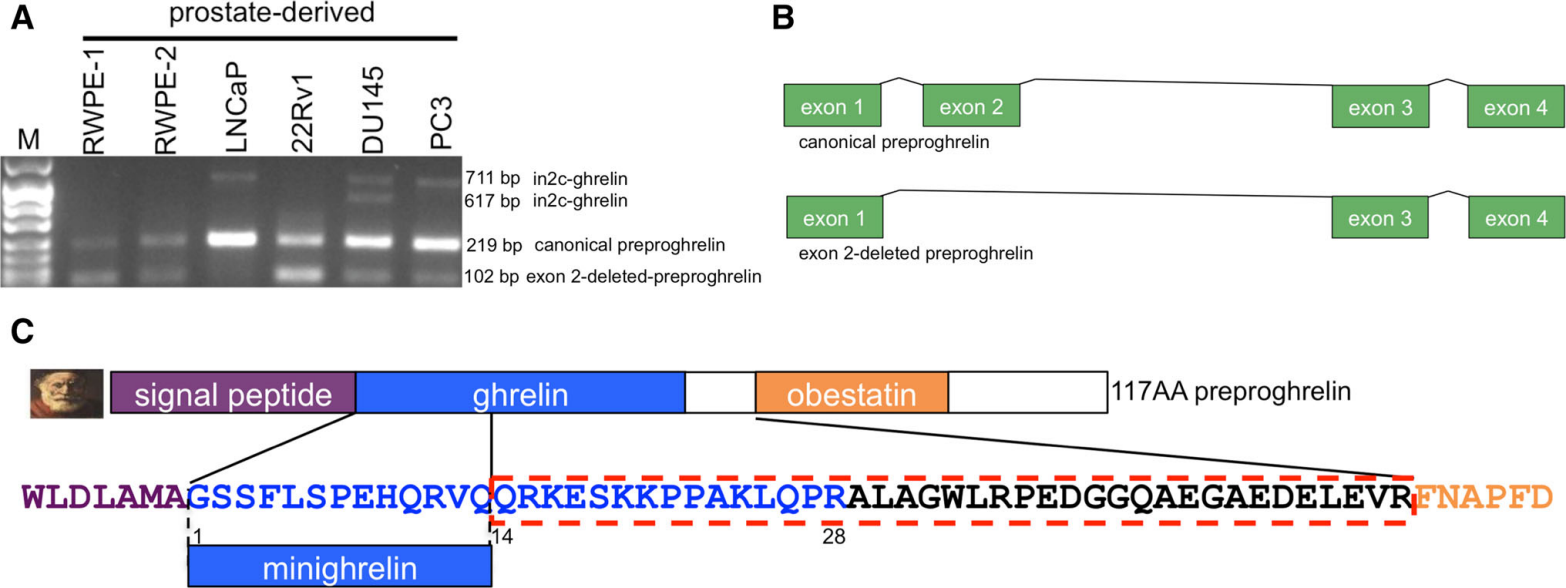
Identification of human exon 2-deleted preproghrelin. **a** Representative RT-PCR products from human normal prostate-derived (RWPE-1, RWPE-2) and prostate cancer-derived (LNCaP, 22Rv1, DU145, PC3) cell lines. Amplicons corresponding to in2c-ghrelin variants (GenBank accession no. EF139854-55), canonical preproghrelin coding exons and the novel exon 2-deleted preproghrelin variant (GenBank accession no. KF921297) are indicated. *M* denotes low-mass DNA molecular weight marker VIII (Roche). **b** Schematic of canonical and exon 2-deleted preproghrelin sequences. Exons and introns are shown as *boxes* and *lines*, respectively. **c** Schematic protein structure of wild-type 117 preproghrelin encoded by exons 1, 2, 3 and 4. The sequence *outlined in red* indicates preproghrelin peptide sequence that is absent in the exon 2-deleted preproghrelin variant. Sequences of the signal peptide (*purple*), minighrelin (*blue*) and obestatin (*orange*) are shown

We recently described the insulin-responsive in2c-ghrelin splice variant in prostate cancer cell lines, [16] and while screening these cells, we identified a novel RT-PCR amplicon (Fig. 2a). This amplicon was cloned from the PC3 prostate cancer cell line (GenBank accession no. KF921297), and from brain and uterus (Online Resource 3). Sanger sequencing revealed that it corresponds to the predicted exon 2-deleted preproghrelin splice variant (Figs. 2b, c). Orthologous full-length exon 2-preproghrelin transcripts were identified in the mouse (GenBank accession no. AB111891) and sheep (GenBank accession no. KF219676) [17]. In1-ghrelin and exon 2-deleted preproghrelin transcripts are present in humans and mice, which represents conservation spanning at least 90 million years. We have cloned exon 2-deleted ghrelin gene-derived transcripts from human, mouse and sheep, and the open reading frame is conserved in multiple species, spanning ~400 million years of vertebrate evolution [31]. This is consistent with, at the very least, mammalian-specific alternative splicing of the ghrelin gene and is suggestive of biologically functional ghrelin gene-derived splice variants with C-terminally truncated ghrelin sequence (minighrelin).

### Minighrelin mimics canonical ghrelin function

The splice variants described here harbour C-terminally truncated ghrelin peptide sequences that retain the first 13 amino acids of the peptide hormone, ghrelin, followed by obestatin sequence. We investigated whether the truncated ghrelin peptide derived from the exon 2-deleted splice variant could mimic the functional effects of canonical ghrelin. This region of the exon 2-deleted prepropeptide retains the active core (the first five amino acids) of ghrelin, which is required for the activation of its cognate receptor, GHSR1a [32, 33], and for octanoylation of the peptide by GOAT [34].

**Fig. 3:**
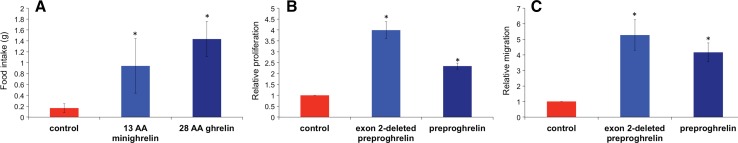
Minighrelin mimics the function of ghrelin. **a** Effects of intraperitoneal (i.p.) administration of synthetic human 28-AA acyl ghrelin or 13-AA acyl minighrelin on short-term food intake in mice. Four-month-old male C57BL/6 mice were injected i.p. with 2 nmol acyl ghrelin (*n* = 6), 2 nmol acyl minighrelin (*n* = 5) or 100 μl vehicle control (sterile saline; *n* = 6). Food intake (standard chow) was measured 4 h after the administration of ghrelin peptides or vehicle. Data points correspond to the mean 4-h food intake (±SEM). *P ≤ 0.05, relative to control (saline). Desacyl ghrelin and desacyl minighrelin had no effect on food intake (data not shown). **b** Effect of exon 2-deleted preproghrelin and canonical preproghrelin overexpression on proliferation of PC3 prostate cancer cells after 60 h (compared to cells expressing vector alone). Data represent the mean ± SEM of three experiments (*n* = 16 per treatment). *P ≤ 0.05, relative to control (empty vector). **c** Effect of exon 2-deleted preproghrelin and canonical preproghrelin overexpression on cell migration in the PC3 prostate cancer cell line after 24 h (compared to cells expressing vector alone). Migration was examined in a Transwell migration assay (xCELLigence, Roche). Data represent the mean ± SEM of three experiments (*n* = 3 per treatment). *P ≤ 0.05, relative to control (empty vector)

We first employed the classical assay for ghrelin function, food intake [3], to determine the effect of the minighrelin peptide sequence on acute appetite stimulation. Synthetic acylated 13-AA ghrelin (minighrelin) peptide stimulated 4-h food intake during the light phase in non-fasted C57BL/6J mice (Fig. 3a) (*P* ≤ 0.05, Mann–Whitney *U* test). Desacyl minighrelin did not stimulate food intake (data not shown).

We next investigated the function of exon 2-deleted preproghrelin in our model assay system, cultured prostate cancer cell lines [16, 35–37]. We employed the PC3 prostate cancer cell line, which expresses exon 2-deleted preproghrelin (Fig. 2a), the processing enzymes necessary to produce mature, acylated ghrelin, the octanoylating enzyme GOAT [36] and the cognate ghrelin receptor, GHSR1a [35]. A similar approach has been used to demonstrate that the in1-ghrelin variant increases proliferation in the MDA-MB-231 breast cancer cell line [15], mirroring the effect of synthetic 28-AA ghrelin peptide treatment [38]. Overexpression of exon 2-deleted preproghrelin (which harbours a 13-AA ghrelin sequence) and full-length preproghrelin (28-AA ghrelin sequence) significantly increased cell proliferation and migration in the PC3 prostate cancer cell line (Fig. 3b, c) (*P* ≤ 0.05, ANOVA with Tukey’s post hoc test). These findings are consistent with our previous studies, which have demonstrated that treatment of prostate cancer cell lines with exogenous 28-AA ghrelin peptide stimulates cell proliferation [35, 37]. Ghrelin stimulates cell migration in cultured cell lines derived from cancers of the brain [39], colon [40] and pancreas [41]. For the first time, we show that canonical preproghrelin and exon 2-deleted preproghrelin also induce cell migration in a prostate cancer cell line. These findings are in agreement with a very recent study showing that a 19-AA putative human in1-ghrelin-derived ghrelin peptide, which harbours the first 13 AAs of ghrelin, acts via the cognate ghrelin receptor, GHSR1a, and is able to mimic the effects of ghrelin [42]. Taken together, these data suggest that naturally occurring ghrelin gene-derived variants coding for a C-terminally truncated ghrelin peptide sequence, minighrelin, are able to mimic the effect of ghrelin.

### Implications of this study

Aided by comparative genomics analysis, we present preliminary evidence of the first conserved ghrelin gene-derived splice variants since the discovery of ghrelin in 1999 [1]. Alternative splicing is a complex event where protein diversity is increased from a relatively small number of genes [43]. Molecular innovation of the ghrelin gene, through alternative splicing, has largely been reported in the literature as species specific. This includes the recently described in2c-ghrelin in humans [16], the mouse-specific ghrelin gene-derived transcript (GGDT) with a novel first exon in intron 2 followed by exon 3 and 4 [44], and a variant with a 5′ truncated exon 3 lacking the N-terminus of obestatin in the red-eared slider turtle [6, 45]. An exon 3-deleted preproghrelin variant with a novel C-terminal region is present in mouse [46] and human [37], but absent in other mammals such as ruminants, where skipping of exon 3 alone would result in a prematurely truncated preproghrelin peptide (unpublished data). It has been proposed that highly conserved alternative splicing corresponds to molecular changes that are tolerated during evolution [47, 48]. Here, we show that skipping exon 2 of the ghrelin gene results in an open reading frame that is highly conserved in vertebrates. While exon 2-deleted preproghrelin produces a C-terminally truncated ghrelin peptide, minighrelin, it is otherwise identical to canonical preproghrelin. We also show that although vertebrate in1-ghrelin sequences have a highly divergent C-terminus, they all harbour an in-frame minighrelin peptide sequence.

The proteolytic cleavage of ghrelin gene-derived peptides remains largely unresolved; however, there are some commonly recognised preprohormone cleavage sites [49]. The obestatin peptide is spanned by an Arg (R) or Gln (Q) in primates [11], and 10 years after its discovery the proteases responsible for its cleavage have not been firmly established. Similarly, minighrelin is spanned by Gln (Q) in all but two species (the common shrew and anole lizard, a discrepancy which may result from genome sequencing errors) and by mono- or dibasic (Arginine and Lysine) amino acid residues in all species except turtles (Online Resource 2). The small size of the putative minighrelin peptide (<1.5 kDa), and difficulty in distinguishing proteolytic cleavage products of canonical ~3.3 kDa ghrelin from minighrelin, has so far yielded mass spectrometry-based detection of minighrelin peptide unsuccessful. Moreover, it is possible that the human exon 2-deleted preproghrelin variant is not cleaved after 13-AA ghrelin peptide sequence and this would give rise to a chimaeric minighrelin–obestatin peptide. Ghrelin and obestatin are multifunctional peptide hormones [12, 50] and chimaeric peptide hormones can have improved, or novel, pharmacological properties compared to the individual peptides alone [51, 52].

It is reasonable to assume that minighrelin is acylated and functional regardless of its processed length. Human exon 2-deleted preproghrelin and in1-ghrelin lack the C-terminal 15 amino acids of the 28-amino acid canonical ghrelin peptide, but are able to mimic the function of ghrelin in vitro. Recent studies suggest that the C-terminal region of ghrelin may be particularly important for stabilising ghrelin in plasma [53, 54]. Therefore, in vivo, the minighrelin peptide might have a shorter half-life than canonical ghrelin and alternative splicing could provide a mechanism for regulating the bioavailability of circulating ghrelin. Nevertheless, even if short ghrelin peptides are less stable in the circulation, ghrelin produced in the stomach binds to its cognate receptor, GHSR1a, on vagal afferent neurons to signal centrally [55, 56] and ghrelin has local (autocrine and paracrine) effects in a number of cell types [13]. Moreover, minighrelin retains the active core of ghrelin [32–34] and it is now firmly established that short synthetic ghrelin peptides have agonist activity [32, 53, 54, 57, 58], albeit with reduced potency compared to full-length ghrelin. Interestingly, short functional ghrelin peptides have been identified in the goldfish (*Carassius auratus*) [5, 59]. We speculate that these short ghrelin peptides in goldfish result from orthologous splice variants of exon 2-deleted preproghrelin or in1-ghrelin. Further proteomic and biochemical analyses are required to characterise variants with minighrelin peptide sequences.

More broadly, it is critical to appreciate the transcriptional complexity of the ghrelin gene locus in normal physiology and disease. Emerging technologies, such as RNA CaptureSeq which allows the detection of low-abundance gene expression in selected loci [60], are likely to greatly advance our understanding of the complex ghrelin gene locus. Since the discovery of ghrelin [1], approximately 8000 articles on ghrelin have been published, with many articles referring to the measurement of serum ghrelin levels and their association with physiology or disease. Discrepancies between studies [4] may be explained partly by the fact that most assays cannot currently discriminate between distinct ghrelin gene-derived peptides. In particular, several studies have relied on sandwich (two-site) ELISAs, with capture antibodies raised against the C-terminal region of the 28-amino acid ghrelin peptide [61, 62]. As these assays cannot detect minighrelin peptides, this would lead to an underestimation of total ghrelin levels. It may be necessary to conduct additional studies under many different physiological and pathophysiological conditions to ensure that the contribution of ghrelin peptides is reliably assessed.

In summary, we provide preliminary evidence that ghrelin gene-derived splice variants encoding C-terminally truncated peptides that retain the active core (the first five amino acids) of ghrelin, termed minighrelins, are conserved in vertebrates. Minighrelin peptide is able to stimulate appetite in mice, and preproghrelin splice variants that encode minighrelin are able to mimic the actions of canonical ghrelin in vitro. These findings add complexity to the study of ghrelin and further impetus for the study of alternative splicing of the ghrelin gene and the function of novel transcripts in diverse species.

## Electronic supplementary material

Below is the link to the electronic supplementary material.

Online Resource 1 Box plot of ghrelin gene exon 2 size variation in vertebrates. Species were grouped according to the UCSC multiway genome subset. The box represents the lower and upper quartile separated by a thick line, which is the median. Circles represent values that are considered to be outliers and may represent sequencing or prediction errors or *bona fide* sequence changes in a species (PDF 100 kb)

Online Resource 2 Multiple sequence alignment of putative vertebrate exon 2-deleted preproghrelin peptides in 77 vertebrate species. The signal peptide (purple), minighrelin (blue) and obestatin (orange) are shown (PDF 142 kb)

Online Resource 3 Identification of human exon 2-deleted preproghrelin in normal tissues. Ethidium-bromide stained agarose gel of RT-PCR amplicons from preproghrelin exon 1 to 3 from an OriGene human tissue Rapid-Scan cDNA panel. The panel consists of normalised cDNA from 48 normal tissues. *M* denotes HyperLadder 50 bp molecular weight marker (Bioline). Tissues expressing exon 2-deleted preproghrelin are indicated in orange. Note that in2c-ghrelin (highlighted in blue) is restricted to male reproductive tissues. NTC = no-template control, where water was substituted for cDNA. 2D +ve cDNA = positive control. PBL = peripheral blood leucocytes (PDF 2050 kb)
