# Supplementary figures and images for: Multi-species sequence comparison reveals conservation of ghrelin gene-derived splice variants encoding a truncated ghrelin peptide
Source: Endocrine. 2016 Jan 20;52:609–17. doi: 10.1007/s12020-015-0848-7 (PMC4879156; doi:10.1007/s12020-015-0848-7)

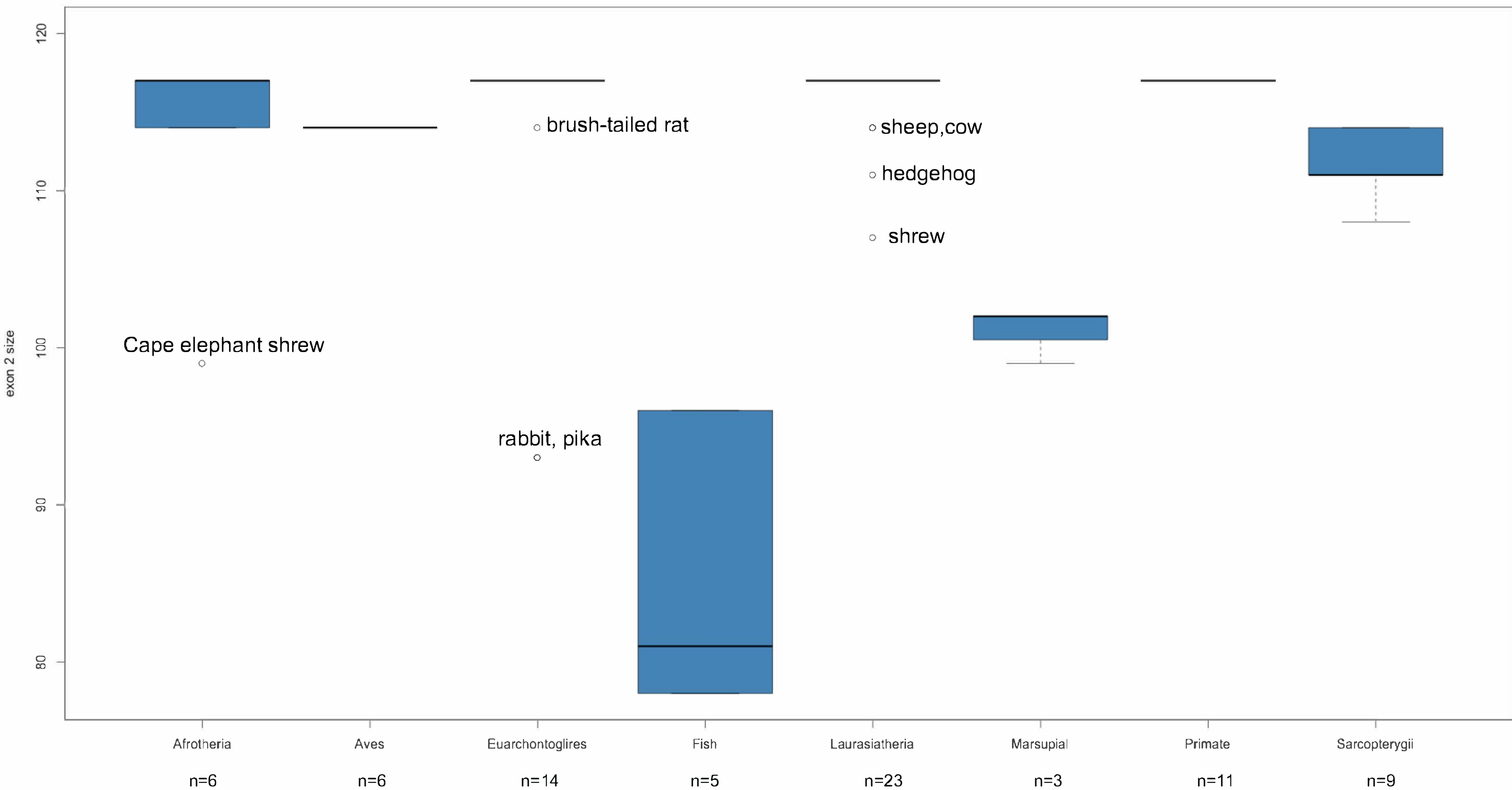

Supplement: Supplementary file 1 — Box plot of ghrelin gene exon 2 size variation in vertebrates. Species were grouped according to the UCSC multiway genome subset. The box represents the lower and upper quartile separated by a thick line, which is the median. Circles represent values that are considered to be outliers and may represent sequencing or prediction errors or bona fide sequence changes in a species (PDF 100 kb) [file 12020_2015_848_MOESM1_ESM.pdf]

# Online Resource 3

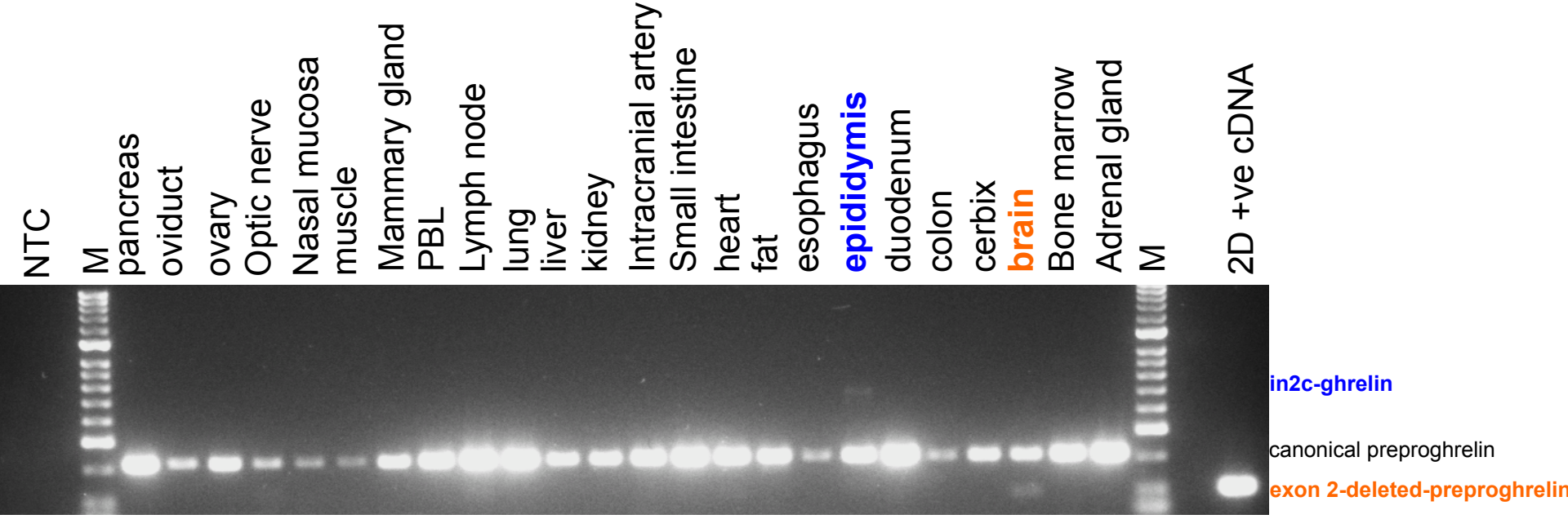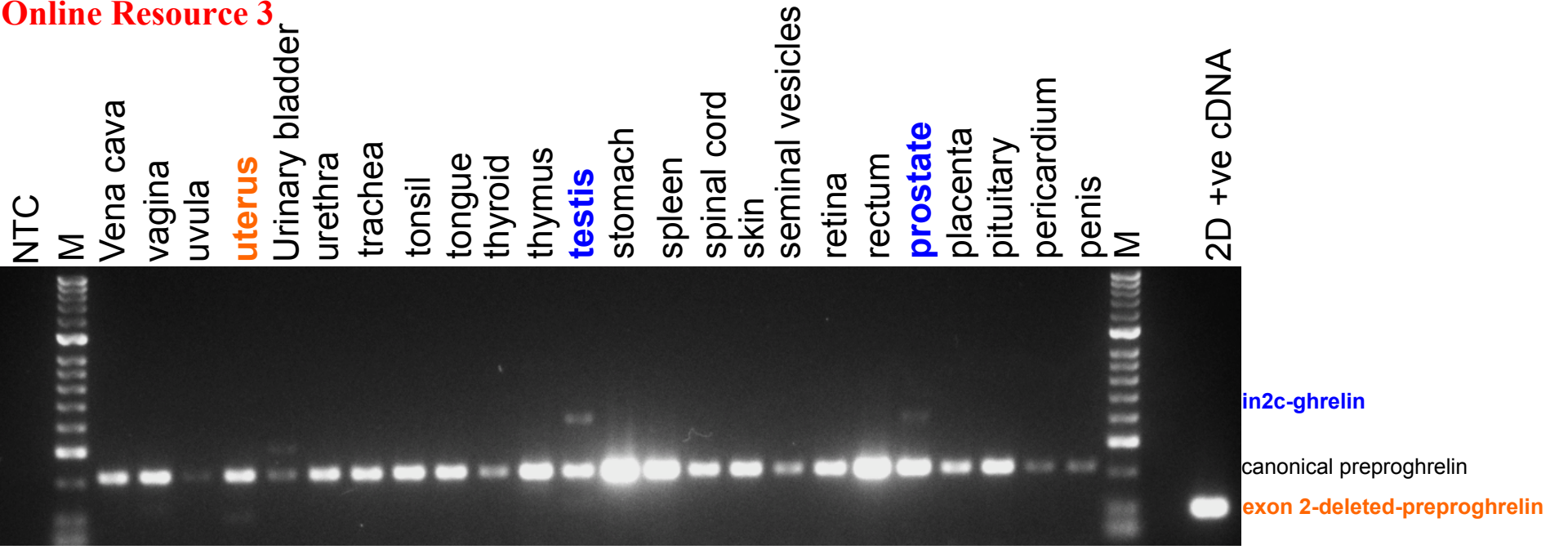

Supplement: Supplementary file 3 — Identification of human exon 2-deleted preproghrelin in normal tissues. Ethidium-bromide stained agarose gel of RT-PCR amplicons from preproghrelin exon 1 to 3 from an OriGene human tissue Rapid-Scan cDNA panel. The panel consists of normalised cDNA from 48 normal tissues. M denotes HyperLadder 50 bp molecular weight marker (Bioline). Tissues expressing exon 2-deleted preproghrelin are indicated in orange. Note that in2c-ghrelin (highlighted in blue) is restricted to male reproductive tissues. NTC = no-template control, where water was substituted for cDNA. 2D +ve cDNA = positive control. PBL = peripheral blood leucocytes (PDF 2050 kb) [file 12020_2015_848_MOESM3_ESM.pdf]
