# Supplementary material for: Multi-species sequence comparison reveals conservation of ghrelin gene-derived splice variants encoding a truncated ghrelin peptide
Source: Endocrine. 2016 Jan 20;52:609–17. doi: 10.1007/s12020-015-0848-7 (PMC4879156; doi:10.1007/s12020-015-0848-7)
Supplement: Supplementary file 2 — Multiple sequence alignment of putative vertebrate exon 2-deleted preproghrelin peptides in 77 vertebrate species. The signal peptide (purple), minighrelin (blue) and obestatin (orange) are shown (PDF 142 kb) [file 12020_2015_848_MOESM2_ESM.pdf]

Online Resource 2

```
human                      1 MPSPGTVCSL-LLLGML--WLDLAMAGSSFLSP-EHQRV--QFNAPFDVGIKLSGVQYQQHSQALGKFLQDILWE-EAKEAP----ADK------ 78 AA
frog                       1 MWSRVFICGV-VSVCLL--WPEAVTAGTSFLSPADMPKS--SVTFPLDINLKMAEKQFQKQKAALQDILLALFSVTPSQDTQ---DGAE------ 81 AA
lizard                     1 MFIRSIIVGT-LLVCSL--WIETTVAGSSFLSP-EQPKM--QFTVPFEIGMKISEAQYKDCEQMLEKILEDILA-------------------- 68 AA
rock dove                  1 MFLRSALLGI-LLSSSL--WTEPSLAGSSFLSP-EYKKL--QFNVPFEIGVRITEEEYQEYRQVLEKMLGDTLGA-SAQATQ----MKN------ 78 AA
chicken                    1 MFLRVILLGI-LLLSIL--GTETALAGSSFLSP-TYKNI--QFNVPFEIGVKITEREYQEYGQALEKMLQDILAE-NAEETR----TKS------ 78 AA
turkey                     1 MFLRLALLGI-LLLSIL--GTETAQAGSSFLSP-AYKNI--QFNVPFEIGVKITEREYQEYGQALEKMLQDIFEE-NAKETQ----TKD------ 78 AA
emu                        1 MFLRGALLVI-LLFSVL--WTETTLAGSSFLSP-DYKKI--QFNVPFEIGVKITEEQYQEYGQMLEKVLGDILEE-NTKETR----MKN------ 78 AA
goose                      1 MFLRGTLLGI-LLFSIL--WTETALAGSSFLSP-EFKKI--QFNVPFEIGVKITEEEYQEYGQTLEKMLQDILEE-NAKETP----VKN------ 78 AA
duck                       1 MFLRGTLLGI-LLFSIL--WTETGLAGSSFLSP-EFKKI--QFHVPFEIGVKITEEEYQEYGQTLEKMLQDILKD-NAKETP----VKS------ 78 AA
American alligator         1 MFLRSALLGI-LLFCIL--YTETTLAGSSFLSP-EYPRT--QCNVPFEIGVKITEGQYQEYGQKLERILEDMLTG-EPKEIQ----VEN------ 78 AA
Chinese alligator          1 MFLRSALLGI-LLFCIL--YTETTLAGSSFLSP-EYPRT--QCNVPFEIGVKITEGQYQEYGQKLERILEDMLTG-EPKEIQ----VEN------ 78 AA
Western painted turtle     1 MFLRSTMLGI-LLICIL--WTETTMAGSSFLSP-EYQNT--QLNVPFEIGVKITEDQYQEYGQVLEKILEDILAE-DTKETRNWHELKHEDVTNL 88 AA
red-eared slider turtle    1 MFLRSTMLGI-LLICIL--WTETTMAGSSFLSP-EYQNT--QLNVPFEIGVKITEDQYQEYGQVLEKILEDILAE-DTKETRNWHELKHEDVTN- 87 AA
green sea turtle           1 MFLRSTMLGI-LLICIL--WTETIMAGSSFLSP-EYQNT--QLNVPFEIGVKITEDQYQEYGQVLEKILEDILAE-DTLETQNWHELKHEDVTN- 87 AA
spiny soft-shell turtle    1 MFLRSTKMGI-LLICIL--WTETVMTGSSFLSP-DYQNT--QLNVPFEIGVKITEDQYQEYQQMLEKILEDVLAE-DTKEIQKLTRAET------ 82 AA
Chinese soft-shell turtle  1 MFLRSTKMGI-LLLCIL--WTETAMTGSSFLSP-EYQNT--QLNVPFEIGVKITEDQYQEYGQVLEKILEDVLAE-DTKEIQTLTRAET------ 82 AA
wallaby                    1 MFPKAALCSL-FLLSVL--WIDVALGGSSFLSP-EHPKT--QFNAPFDIGIKVAEAQYQQYGRALEKVLQEILLE-KNQGNI----GEN------ 78 AA
Tasmanian devil            1 MLPKVAIGSL-LLLSVL--WMDVAWAGSSFLSP-EHPKT--QFNAPFDIGIKVAEAQYRQYGHALEKVLQEILLE-ENQGNT----GEN------ 78 AA
opossum                    1 MIPKVAICSL-LLFSVL--WMDVALAGSSFLSP-EHPKT--QFNAPFDIGIKVAEAQYQQYGHALEKVLQEILLE-ENQGPT----GGK------ 78 AA
guinea pig                 1 MTLAGTICSL-LLLSVL--WMELAMAGASFRSP-EHHSA--QFNVPLDIGVKLSGAQYQQHSQALGKLLQAVLGE-EAEEAP----ADK------ 78 AA
tenrec                     1 MLSRGTICVL-LLLSVL--WVDLTRAGSSFLSP-GHPKV--QFNVPFDIGIKVSVAQYGEHGRALDKFLQDILWE-GNQEAP----TDK------ 78 AA
cow                        1 MPAPWTICSL-LLLSVL--CMDLAMAGSSFLSP-EHQKL--QFNAPFNIGIKLAGAQSLQHGQTLGKFLQDILWE-EAEETL----ANE------ 78 AA
sheep                      1 MPAPRTIYSL-LLLSLL--WMDLAMAGSSFLSP-EHQKL--QFNAPFNIGIKLSGAQSLQHGQTLGKFLQDILWE-EAEETL----ADE------ 78 AA
Cape elephant shrew        1 MLSQGTICSL-LLLSTL--CLDLAAAGSSFLSP-EHQKV--QFNVPFDVRIKLSGAQYQQHGHALGKFLQDILWE-DVKDNP----TNK------ 78 AA
Chinese tree shrew         1 MLSPRTICAL-LLLSML--WVDSAAAGSSFLSP-EHQKA--QFNTPFDVGIKLSGAQFQQHGQALGKFLQDILWE-EAEDAA----ADN------ 78 AA
Cape golden mole           1 MFSSGTIYSL-LLLSVL--WVDFTVAGSSFLSP-EHQKV--QFNAPFDVGIKLSGAQYKQHGGALGKFLQDILWE-DDKEVP----SDN------ 78 AA
Aardvark                   1 MLSLGTICSL-LLFSVL--WADLAMAGSSFLSP-EHQKV--QFNVPFDIGIKLSGPQYRHHGQALEKFLQDILWE-DAKEAL----NNK------ 78 AA
alpaca                     1 MPSPGTICGL-LLLSVT--WVDVAMAGSSFLSP-ERQRA--QFNAPFDVGIKLSGAQSHQHGQTLGNFLQDILWE-EANEAP----ANE------ 78 AA
Batrican camel             1 MPSPGTICGL-LLLSVI--WVDVAMAGSSFLSP-ERQRA--QFNAPSDVGIKLSGAHSHQHGQTLGKFLQDILWE-EANEAP----ANE------ 78 AA
black flying fox           1 MPSLGTICSL-LLLSVL--WVDLAMAGSSFLSP-EHQKV--QFNTPFDVGIKLSGAQDRRPGQVLEKFLQDVLWE-EASEVL----ADK------ 78 AA
large flying fox           1 MPSLGTICSL-LLLSVL--WVDLAMAGSSFLSP-EHQKV--QFNTPFDVGIKLSGAQDRRPGQVLEKFLQDVLWE-EASEVL----ADK------ 78 AA
rabbit                     1 MLSAGTACSL-LLLSVL--WVDVAMAGSSFLSP-EHQKA--QFSAPFDVGIQLSGAQYQQHGRALGKILQDILRE-ETKEAS----ADQ------ 78 AA
pika                       1 MLSARTTCSL-LLLSLL--WVDMAMAGSSFLSP-EHQKA--QFSAPFDVGIQLSGAQYQQHGQALSKILQDILRE-QTKETS----ADQ------ 78 AA
dolphin                    1 MPSPGTVCSL-LLFSVL--WVDLAMAGSSFLSP-EHQKV--QFSAPFDVGIKLSGAQSHQHGQTLGKFLQDVLWE-DASETP----AHK------ 78 AA
killer whale               1 MPSPGTVCSL-LLFSVL--WVDLAMAGSSFLSP-EHQKV--QFSAPFDVGIKLSGAQSHQHGQTLGKFLQDVLWE-DASETP----AHK------ 78 AA
dog                        1 MPSLGTMCSL-LLFSVL--WVDLAMAGSSFLSP-EHQKL--QFNAPFDVGIKLSGPQYHQHGQALGKFLQEVLWE-DTNEAL----ADE------ 78 AA
cat                        1 MPSPGTVCSL-LLFSML--WADLAMAGSSFLSP-EHQKV--QFNAPFDVGIKLSGAQYHQHGQALGKFLQDVLWE-EADEVL----ADE------ 78 AA
Pacific walrus             1 MPSPGTLCSL-LLFTVL--WMDLAMAGSSFLSP-EHQKA--QFDVPFDVGIKLLGAQYHQHSQALGKFLQDILWE-EANEAL----ADE------ 78 AA
Weddel seal                1 MPSLGTLCSL-MLFTVL--WMDLAMAGSSFLSP-EHQKA--QFNVPFDVGIKLLGAQYHQHSQALGKFLQDILWE-EANEAL----ADE------ 78 AA
panda                      1 MPSLGTLCSL-LLFSVL--WMDLAMAGSSFLSP-EHQKV--QFNAPFDVGIKLSGAQYQEHGQALGKFLQDILWE-EANEAL----ADE------ 78 AA
ferret                     1 MPSLGTLCSL-LLFSVL--WMDLALAGSSFLSP-EHQKV--QFNTPFDVGIKLSGAQYHQHGQALGKFLQDILWE-EANEAL----EDK------ 78 AA
orangutan                  1 MPSPGTICSL-LLLGML--WLNLAMAGSSFLSP-EHQRV--QFNAPFDVGIKLSGVQYQQHSQALGKFLQDILWE-KAKEAP----ADK------ 78 AA
rhesus                     1 MPSPGNVCSL-LLLGML--WLDLAMAGSSFLSP-EHQRA--QFNAPFDVGIKLSGVQYQQHSQALGKFLQDILWE-EAKEAP----ADK------ 78 AA
baboon                     1 MPSPGNVCSL-LLLGML--WLDLAMAGSSFLSP-EHQRA--QFNAPFDVGIKLSGVQYQQHSQALGKFLQDILWE-EAKEAA----ADK------ 78 AA
green monkey               1 MPSPGNICSL-LLLGML--WLDLAMAGSSFLSP-EHQRV--QFNAPFDVGIKLSGVQYQQHSQALGKFLQDILWE-EAKEAP----ADK------ 78 AA
chimp                      1 MPSPGTVCSL-LLLGML--WLDLAMAGSSFLSP-EHQRV--QFNAPFDVGIKLSGVQYQQHSQALGKFLQDILWE-EAKEAP----ADK------ 78 AA
gibbon                     1 MPSPGTVCSL-LLLGML--WLDLAMAGSSFLSP-EHQRV--QFNAPFDVGIKLSGVQYQQHSQALGKFLQDILWE-EAKEAP----ADK------ 78 AA
gorilla                    1 MPSPGTVCSL-LLLGML--WLDLAMAGSSFLSP-EHQRV--QFNAPFDVGIKLSGVQYQQHSQALGKFLQNILWE-EAKEAP----ADK------ 78 AA
marmoset                   1 MPSPKTICGL-LLLSVL--SLDLAMAGSSFLSP-EHQRI--QFNAPFDVGIKLSGVQYQQHSQALGKFLQDILWK-EAKEAP----TDK------ 78 AA
squirrel monkey            1 MPSPKTICSL-LLLCVL--WLDLAMGGSSFLSP-EHQRI--QFNAPFDVGIKLSGVQYQQHSQALGKFLQDILWE-EAKEAP----ADK------ 78 AA
horse                      1 MPSRGTICSL-LLLSVL--WVDLTMAGSSFLSP-EHHKV--QFNAPFDVGIKLSGAQYHQHSQALGTFLQDILWE-EANEAP----DDR------ 78 AA
white rhinoceros           1 MPSLGTIRSL-LLLSVL--WVDLTMAGSSFLSP-EHQKV--QFNAPFDVGIKLSGAQYHQHSQSLGKFLQDILWE-EANEDQ----ADK------ 78 AA
lesser Egyptian jerboa     1 MLSAGTFYSL-LLLSVL--WVDLAMAGSSFLSP-EHQKA--QFNAPLDVGVRLSGAQYQQHERALGKFLQDILWE-EVKEAT----ADK------ 78 AA
squirrel                   1 MFSAGTICSL-LLLSVL--WVDVAMAGSSFLSP-EHQKA--QFNAPFDMAIKLFGAQYQQHSRALRKFLQDILWE-EAKEAL----ADQ------ 78 AA
bushbaby                   1 MLSLGTICSL-LLLSVL--WVDVAMAGSSFLSP-DHQKI--QFNSPLDVGIKLSGAQYQQHSQALGKFLQDILWE-EAK--------------- 72 AA
brush-tailed rat           1 MISAGTLCSL-LLLGVL--WVDLAMAGSSFLSP-EHQRA--QFNAPFDVGIKLSGPQYQQHGQALGKFLHDVL-E-EAK--------------- 71 AA
naked mole rat             1 MISARTLCSL-LLLSVL--WVDLAMAGSSFLSP-GHQKA--QFNAPFDVSIKLSGAQYQQHCQALEKFLQDILGE-EAKEAP----ADK------ 78 AA
chinchilla                 1 MISAGTLCSL-LLLSVL--WVDLAMAGSSFLSP-EHRKA--QFNAPFDVGIKLSGAQYQQHGQALGKFLQDILGE-EAKEAP----ADK------ 78 AA
rat                        1 MVSSATICSL-LLLSML--WMDMAMAGSSFLSP-EHQKA--QFNAPFDVGIKLSGAQYQQHGRALGKFLQDILWE-EVKEAP----ANK------ 78 AA
mouse                      1 MLSSGTICSL-LLLSML--WMDMAMAGSSFLSP-EHQKA--QFNAPFDVGIKLSGAQYQQHGRALGKFLQDILWE-EVKEAP----ADK------ 78 AA
prarie vole                1 MPSSGTICSL-LLLSVL--WMDVAMAGSSFLSP-EHQKA--QFNAPFDVGIKLSGAQYQQHGRALGKFLQDILWE-EVKEAP----ADK------ 78 AA
Chinese hamster            1 MLSSGTICSL-LLLSVL--WMDVAMTGSSFLSP-EHQKA--QFNAPFDVGIKLSGAQYQQHGQALGKFLQDILWE-EVKEAP----ADK------ 78 AA
golden hamster             1 MLSSGTICSL-LLLSVL--WMDVAMTGSSFLSP-EHQKA--QFNAPFDVGIKLSGAQYQQHGRALGKFLQDILWE-EVKEAP----ADK------ 78 AA
African elephant           1 MLSTGTLCSL-LLLSVF--WVDFAMAGSSFLSP-KNQKL--QFNAPFDIGIKLSGAQYQQHGWALGKFLQDILWE-EAKEAP----ADK------ 78 AA
manatee                    1 MLSTGTICSL-LLLSVL--WVDLAMAGSSFLSP-KHQKV--QFNAPFDVGIKLSGAQYQQHGRALGKFLQDILWE-EAKEAP----ADK------ 78 AA
pig                        1 MPSTGTICSL-LLLSVL--LADLAMAGSSFLSP-EHQKV--QFNAPCDVGIKLSGAQSDQHGQPLGKFLQDILWE-EVNEAP----ADK------ 78 AA
Brandt’s bat               1 MPSPGTICSL-LLLSVL--WVDLAMAGSSFLSP-EHQKA--QFNAPFDVGIKLSGAQSHWHGQALGKFLQDMFWE-EANEVP----ADK------ 78 AA
David’s Myotis bat         1 MPSPGTICSL-LLLSVL--WVDLAMAGSSFLSP-EHQKA--QFNAPFDVGIKLSGAQSHWHGQALGKFLQDMFWE-EANEVP----ADK------ 78 AA
little brown bat           1 MPSPGTICSL-LLLSVL--WVDLAMAGSSFLSP-EHQKA--QFNAPFDVGIKLSGAQSHWHGQALGKFLQDMFWE-EANEVP----ADK------ 78 AA
big brown bat              1 MPSPGTICSL-LLLSVL--WVDLAMAGSSFLSP-EHQKV--QFNAPFDVGIKLSGAQSHWHGQALGKFLQDMFWE-EANEAP----ADK------ 78 AA
shrew                      1 MRSLAPACCL-LLLGAL--WAERAAAGSSFLSP-EHHKG--PFNAPFDVGIKLSGVQYHQHGQALSQFLQDVLWE--ANDVP----EDK------ 77 AA
hedgehog                   1 MLSPAAACRL-LLLGAL--CMDLAAAGSSFLSP-EHQKG--QFSAPFDVGLRLSGAQYEQHGEALREILQDVLWE-EARGSP----HDK------ 78 AA
goldfish                   1 MPLRRRASHMFVLLCALSLCVESVKGGTSFLSP--AQKP--QMSAPFELSVSLSEAEYEKYGPVLQKVLVNLLGD-----SP----LEF------ 76 AA
Atlantic salmon            1 MLLKRNTGLMILMLCTLALWAKSVSGGSSFLSP-SQKPQVRQIKAPFEMGITMSEEEFQEYGAVLQKILQDVLGDTATAE--------------- 79 AA
Rainbow trout              1 MPLKRNTGLMILMLCTLALWAKSVSAGSSFLSP-SQKPQVRQIKAPFEMGITMSEEEFQEYGAVLQKILQDVLGDTATAE--------------- 77 AA
tilapia                    1 MLLKRNTCLLAFLLCSLTLWCKSTSAGSSFLSP--SQKP--QLSAPFEIGVTLRAEDLADYIVELQEIVQRLLGNTETAERP----SPR------ 81 AA
black seabream             1 MFLKRNTYLLVFLFCSLTLWCKSTSAGSSFLSP--SQKP--QISAPFEIGISMTEEDYDEYGVVLQEIIQRLLGGTEAAEGP----PQL------ 81 AA
```
